# Utility of Abdominal Radiographs After Posterior Spinal Fusion for Neuromuscular Scoliosis †

**DOI:** 10.3390/jcm14010278

**Published:** 2025-01-06

**Authors:** Tyler A. Tetreault, Rachel Lai, Tiffany N. Phan, Kenneth D. Illingworth, David L. Skaggs, Tishya A. L. Wren, Lindsay M. Andras

**Affiliations:** 1 Jackie and Gene Autry Children’s Orthopedic Center, Children’s Hospital Los Angeles, Los Angeles, CA 90027, USAlandras@chla.usc.edu (L.M.A.); 2 Department of Orthopaedics, Cedars-Sinai Medical Center, Los Angeles, CA 90048, USA

**Keywords:** neuromuscular scoliosis, postoperative management, complications, postoperative ileus, abdominal radiographs, radiation exposure

## Abstract

**Background/Objectives:** Postoperative ileus, the temporary cessation of gastrointestinal motility leading to accumulation of fluid and gas in the bowel, is a common complication following posterior spine fusion (PSF) in patients with neuromuscular scoliosis (NMS). Abdominal radiographs (KUBs) are often ordered to differentiate between ileus and mechanical obstruction but expose patients to radiation, add cost, and may lead to unnecessary work up. The aim of this study was to determine how often KUBs led to a change in treatment after PSF in patients with NMS. **Methods:** A retrospective review was conducted of NMS patients with ≥2-year follow-up treated with PSF between January 2014 and December 2019 at a tertiary pediatric hospital. **Results:** Of the 133 patients (age 13.7, SD 2.6 years; preoperative curve magnitude 82.8, SD 23.0 degrees; follow-up 44.7, SD 15.4 months), 22.6% (30/133) underwent KUB imaging due to abdominal pain, distension, or delayed return of bowel function. In total, 93.3% (28/30) of the KUB imaging was consistent with ileus. One KUB study resulted in a gastroenterology consultation and bowel cleanout, and one raised concerns regarding possible pneumatosis of the colon, prompting a pediatric surgery consultation and exploratory laparotomy which was unremarkable. **Conclusions:** KUBs were performed in nearly 25% of NMS patients during the acute postoperative period, but rarely provided useful diagnostic information leading to changes in management. In the presence of postoperative abdominal distension, feeding intolerance, and delayed return of bowel function, we advocate for continuing conservative measures until bowel function returns, reserving abdominal radiographs for patients with a worsening exam despite bowel rest or additional causes for concern.

## 1. Introduction

Neuromuscular scoliosis (NMS) is defined as a spinal deformity that develops from a variety of etiologies secondary to abnormal brain, spinal cord, or muscle function. Regardless of the underlying condition, spinal curves are typically progressive and poorly controlled with non-surgical methods [1,2,3,4,5,6,7]. Posterior spinal fusion (PSF) is often indicated to prevent further progression, correct pelvic obliquity and achieve improved seating balance, and improve patient and caregiver quality of life [8,9,10]. Complication rates after PSF for NMS patients are higher than for those with idiopathic scoliosis, with overall complication rates of 24–41% in recent studies [11,12,13,14]. Common postoperative complications in NMS patients are infection, prolonged intensive care and hospital stays, respiratory compromise, and ileus [15].

Postoperative ileus, the temporary cessation of gastrointestinal motility that leads to accumulation of fluid and gas within the bowel, is an often encountered complication after spinal fusion surgery [16,17]. A diagnosis of NMS is a known risk factor for developing postoperative ileus [18]. Rates of gastrointestinal complications and postoperative ileus in the NMS population are reported at 4.5% to 8% [12,19,20]. Though typically managed conservatively, the presence of postoperative ileus can lead to significantly longer hospital stays and the potential need for advanced nutrition support [20,21]. In addition, the clinical presentation of ileus, which includes abdominal pain, distension, vomiting, anorexia, or delayed passage of flatus, overlaps with that of more serious gastrointestinal pathologies, such as acute colonic pseudo-obstruction (Ogilvie syndrome) or superior mesenteric artery syndrome, both of which have been reported in adolescents following surgery for scoliosis [22,23,24,25]. These and other causes of bowel obstruction can lead to bowel ischemia, perforation, or other catastrophic sequelae if not properly diagnosed and managed. Abdominal radiographs (KUBs) are often recommended to differentiate between ileus and mechanical obstruction even though such studies are poor at distinguishing between these pathologies [26]. The widespread use of KUBs may expose patients to unnecessary radiation, add cost to their postoperative care, and lead to further subsequent work up.

The purpose of this study is to examine the utility of KUBs after posterior spinal fusion for neuromuscular scoliosis. Specifically, the study aims to investigate the prevalence of serious gastrointestinal pathology in this population and evaluate the utility of postoperative KUBs in changing patient management.

## 2. Materials and Methods

After obtaining institutional review board approval, we retrospectively reviewed the charts of consecutive patients who underwent PSF for NMS at a single institution between 1 January 2014 and 31 December 2019 and had at least 2 years of postoperative follow-up. All patients who did not have adequate follow-up were excluded.

All subjects underwent instrumented posterior spinal fusion with pedicle screw fixation. A preoperative nutrition consultation and preoperative nutrition labs were not routinely performed. Postoperatively, patients were admitted to the pediatric intensive care unit (ICU) for monitoring. Patients remained nil per os (NPO) with maintenance intravenous (IV) fluids until bowel sounds returned. Those with oral nutritional intake then resumed a clear liquid diet, which was slowly advanced to their home diet as tolerated, while others received gastrostomy tube (G-tube) feeds which were started slowly and gradually advanced, typically starting with a continuous rate of 30cc per hour. Once deemed medically stable by the ICU team, patients were transferred to the orthopedic inpatient ward. Pediatric hospitalists were consulted regarding the co-management of medical comorbidities when deemed appropriate.

Postoperative ileus was diagnosed clinically for patients with abdominal pain, distension, vomiting, and delayed passage of flatus or stool. These patients were managed with bowel rest and IV fluids. Nasogastric tubes were placed for gastric decompression when needed, and in cases with preexisting malnutrition or persistent ileus, parenteral nutrition was started until feeds could be resumed.

Charts were reviewed for demographic information (age, sex, body mass index, and type of neuromuscular diagnosis), clinical characteristics (G-tube dependence, ambulatory status, and preoperative curve magnitude), surgical data (surgical time, curve correction, and estimated blood loss), and additional postoperative interventions, including obtaining KUB studies, pediatric surgery consultations, and additional invasive procedures. The results of these studies and any changes to the postoperative course were recorded.

Descriptive statistics were used to summarize the demographics and clinical characteristics of all subjects. Mean and standard deviation are reported for continuous variables, while counts and percentages are reported for categorical variables. Group demographics (age, sex, underlying neuromuscular diagnosis, nutrition status, ambulatory status, and preoperative curve magnitude) and surgical variables (surgical time, curve correction, and estimated blood loss) were compared between patients who did and did not receive a postoperative KUB using *t*-tests, Fisher’s exact test, and logistic regression analysis. All outcome variables were considered significant if they had a *p* value < 0.05. All of the statistical analysis was performed using STATA 14.0 (College Station, TX, USA: StataCorp LP).

## 3. Results

One hundred and thirty-three patients (57.1% male) with a mean ± standard deviation (SD) age of 13.7 ± 2.6 years (range 9–18 years) met the inclusion criteria. Mean follow-up was 44.7 ± 15.4 months (24–86 months). Demographic data and underlying neuromuscular diagnoses are detailed in Table 1. The preoperative body mass index (BMI) averaged 18.3 ± 4.4 kg/m^2^. Preoperatively, 62 patients (46.6%) received nutrition via gastrostomy tube and 97 patients (72.9%) were non-ambulatory. The average preoperative major Cobb angle measured 82.8 ± 23.0 degrees (36–153 degrees) with an average correction of 55.7 ± 18.7 degrees. The mean length of surgery was 392 ± 129 min, and estimated blood loss (EBL) was 779 ± 460 mL.

Thirty patients (22.6%) received postoperative abdominal radiographs for abdominal pain, distension, and/or vomiting [Table 2]. The median day on which the KUB was performed was postoperative day #2 (range: day #0–4). Fifteen of thirty (50%) studies were reported as consistent with postoperative ileus without additional intra-abdominal pathology and required no change in the clinical management of ileus. Thirteen studies (43.3%) demonstrated nonspecific findings of bowel dilation. These patients underwent continued conservative management and bowel function returned, thus confirming the clinical diagnosis of ileus. Among the 13 patients, pediatric surgery was consulted for one patient where obstruction could not be ruled out based on the KUB findings. This surgery consultation resulted in additional serial abdominal radiographs and close abdominal examinations without changes to conservative management for ileus. Ultimately, a diagnosis of ileus was confirmed with the uneventful return of bowel function.

**Figure 1:**
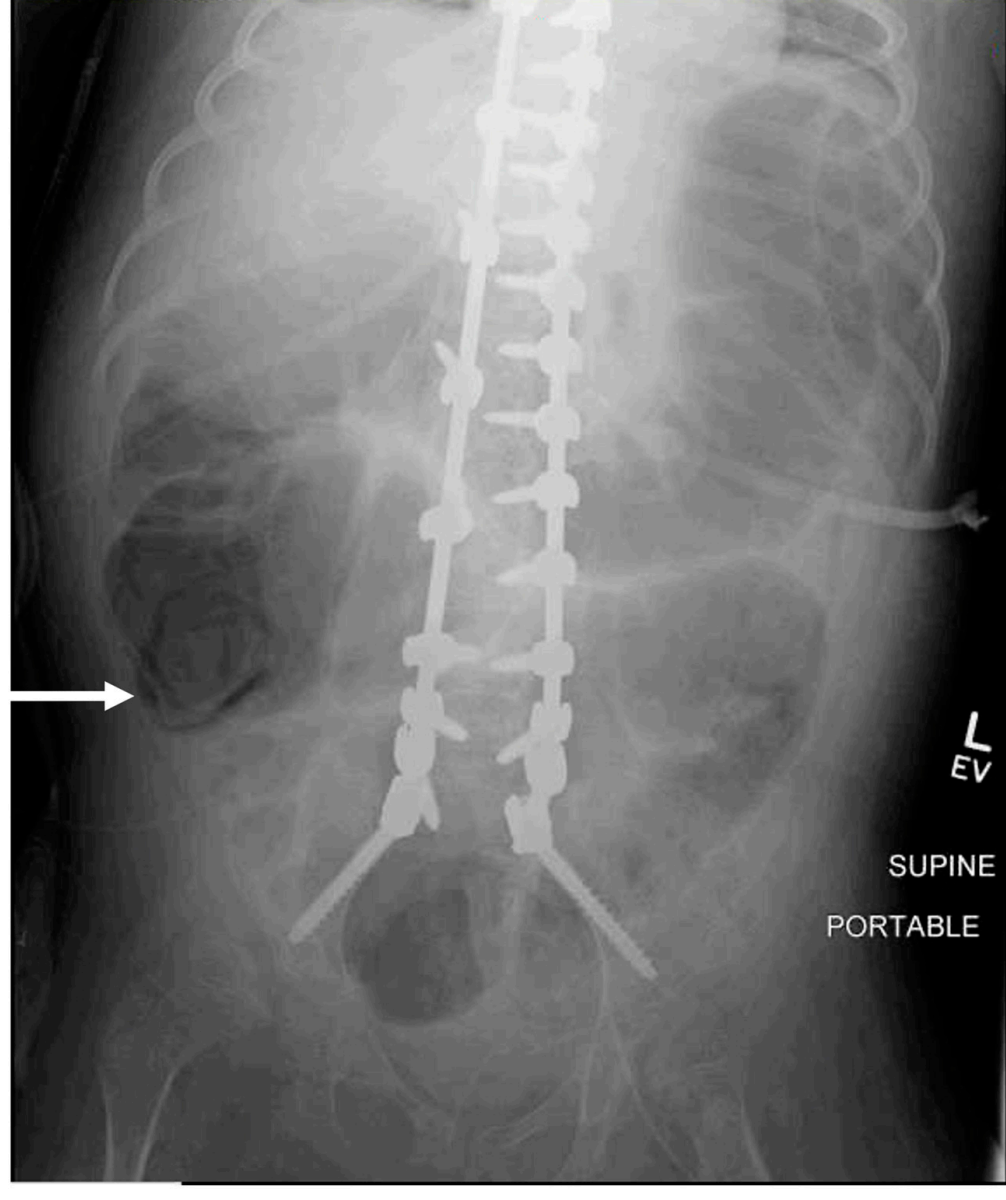
A supine AP abdominal radiograph of a nine-year-old male who underwent PSF for neuromuscular scoliosis was performed for worsening abdominal distension on postoperative day #2. The white arrow demonstrates the area concerning for possible pneumatosis coli, or free air within the wall of the colon.

Two of thirty patients (6.7%) had notable KUB findings. One patient with a history of chronic constipation had an incidental finding of fecal impaction for which the gastroenterology team was consulted, and a bowel cleanout regimen including daily enemas was initiated prior to discharge. The other KUB study reported a concern for pneumatosis of the colon [Figure 1]. Pediatric surgery was consulted and, as this could not be excluded, the patient underwent an exploratory laparotomy due to a concern for bowel ischemia and/or perforation. The intraoperative evaluation did not show any concerning findings and the abdomen was closed primarily and treated as an ileus. Overall, twenty-nine (96.7%) of the thirty patients had postoperative abdominal radiographs performed that resulted in no significant change in management. Furthermore, in the case that the patient had a surgery performed, the findings were unremarkable.

Demographic and surgical characteristics were assessed between groups that did and did not receive a postoperative KUB [Table 3]. There was no significant difference between the groups with regard to age, sex, nutrition status, preoperative BMI, preoperative curve magnitude, magnitude of curve correction, or EBL. In comparison to CP, underlying neuromuscular diagnosis did not portend a greater odds of receiving a postoperative KUB for muscular dystrophy (OR 0.51 [95% CI 0.13–1.96; *p* = 0.328), spina bifida (OR 0.91 [95% CI 0.17–4.91]; *p* = 0.910), or spinal muscular atrophy (OR 0.91 [95% CI 0.17–4.91]; *p* = 0.910). While there was a trend towards an increased incidence of obtaining abdominal films in non-ambulatory patients (86.7% versus 68.9% in ambulatory patients), this did not reach statistical significance (*p* = 0.064). Patients who received a postoperative KUB had a significantly greater mean OR duration of 450 ± 117 min versus 375 ± 128 min for patients who did not receive a KUB (*p* = 0.005).

## 4. Discussion

The utility of postoperative KUBs after spinal fusion for NMS is low. Most patients with abdominal pain and distension, feeding intolerance, and delayed flatus have developed postoperative ileus rather than a bowel obstruction. Postoperative KUBs are not a sensitive test to distinguish between the two processes, can create more confusion, and lead to additional diagnostic studies or invasive procedures. Ultimately, patients who receive a postoperative KUB rarely demonstrate serious gastrointestinal pathology and rarely require any change in postoperative management.

Given the increased hospital costs and length of stay for patients who develop postoperative ileus, investigators have sought to quantify the prevalence of and risk factors for ileus after spine surgery. Reported rates of postoperative ileus after PSF for patients with NMS vary from 4.5 to 8.2% [12,13,19,20]. While most of these studies are small, a large retrospective study by Lee et al. [12] of 2856 patients with CP who underwent spinal fusion reported the development of postoperative ileus in 8.2% of the cohort. In the present study, 28 of 133 (21.1%) patients developed postoperative ileus as diagnosed by clinical exam and abdominal radiographs. It is possible that this underestimates the true occurrence of postoperative ileus in this cohort, as ileus can also be diagnosed on clinical findings alone without abdominal radiographs, though this is often poorly documented and not effectively captured in this retrospective review. Most research regarding the prevention of postoperative ileus derives from general surgery, where intra-abdominal procedures and the development of postoperative ileus are much more common. Recommended approaches to help prevent ileus include pharmacologic treatment with pro-motility agents and limiting narcotic use [27]. Furthermore, the rate of early enteral feeding has been shown to decrease the rate of postoperative ileus after abdominal surgery and decrease length of stay [28]. Despite this, because many of our neuromuscular patients cannot communicate hunger or abdominal discomfort, feeds are held in efforts to prevent postoperative emesis and the risk of aspiration. The role of early enteral feeding in preventing postoperative ileus after pediatric spine surgery warrants further review.

An understanding of the risk factors for the development of postoperative ileus may allow for greater confidence in providing a clinical diagnosis and minimize postoperative KUB use. In a retrospective study of 91 patients with NMS, Jalanko et al. [20] identified increased curve rigidity, a greater preoperative curve magnitude, the presence of intraoperative neuromonitoring alerts, and the use of intravenous pain medications for greater than five days postoperatively as positive risk factors for the development of postoperative ileus. In this study, patients who received a postoperative KUB had a significantly greater OR duration than those that did not, with a mean difference in OR time of 75 min. It is possible that the additional anesthetic dose may predispose patients to the development of postoperative ileus, or that the additional OR time reflects an increased medical or surgical complexity that is not captured by demographic factors such as curve magnitude, G-tube status, or ambulatory status, which were not significantly different between the groups.

The purpose for obtaining a postoperative KUB in this study population was to rule out obstruction in patients with abdominal distension, emesis, and absence of flatus. While ileus is much more likely, obstructive pathology can occur after spinal fusion, and a physical exam alone cannot definitively distinguish between the two processes [22,23,24,25]. However, the KUB is a poor test to differentiate ileus from obstruction. Frager et al. [29] found that, while a CT scan was 100% sensitive and specific in differentiating between the two pathologies, clinical examination and abdominal radiographs were only 19% sensitive and most frequently nondiagnostic, causing only additional confusion. Additional studies have shown that the sensitivity of a KUB in patients with suspected obstruction is only 70–75%, faring worse than both a CT scan and ultrasound in providing an accurate diagnosis [30,31]. In the present study, KUBs provided an accurate diagnosis in approximately half of patients with postoperative ileus. The remainder reported nonspecific findings, which only led to further diagnostic testing and additional exposure to radiation. Ultimately, management relied on continued clinical observation and bowel rest, and the resolution of symptoms confirmed the diagnosis of ileus.

Diagnostic tests with poor specificity risk false positive findings. In this study, one patient with a history of gastrostomy tube dependence, tracheostomy, and severe developmental delay received a KUB that reported colonic pneumatosis. This nonspecific finding can present with bowel obstruction as well as postoperative ileus. Ultimately, the patient underwent an exploratory laparotomy with pediatric surgery, and there was no evidence of bowel ischemia or obstruction at the time of surgery. The patient was treated with 7 days of bowel rest and prophylactic antibiotics, after which repeat abdominal radiographs demonstrated the resolution of pneumatosis, and feeds were restarted via the patient’s gastrostomy tube without further complication. An initial treatment of ileus without additional radiographs could have led to the same clinical result and avoided an unnecessary surgical procedure.

In addition, abdominal radiographs expose patients to additional radiation and potentially increase cancer risk [32]. For adolescents with idiopathic scoliosis, increased radiation from serial radiographs has been associated with increased cancer mortality compared to non-affected peers [33]. This risk may be even greater for patients with NMS who have additional medical conditions that may require frequent diagnostic imaging. While the typical radiation dose from an abdominal radiograph of 0.6 millisieverts (mSv) is well below the accepted level of 100 mSv, at which there is strong evidence to connect radiation exposure with adverse events, there is not a known threshold below which radiation exposure is considered completely safe [34,35].

A limitation of this study is that, due to the retrospective design, we were unable to fully quantify the prevalence of postoperative ileus in patients with NMS. Patients with postoperative ileus that did not receive abdominal radiographs were not accounted for as this is often poorly documented during the inpatient stay. Therefore, we cannot accurately assess the difference in outcomes in the management of ileus for patients that did or did not receive abdominal radiographs. In addition, due to our heterogenous patient population, the modest sample size, the overall low prevalence of some neuromuscular disorders, and the rarity of obstructive GI processes, we were potentially underpowered to assess for risk factors for the development of postoperative ileus or a more serious GI pathology in this cohort. Such information could more effectively inform providers as to the risk of these complications and guide decision-making on whether a postoperative KUB will change treatment.

## 5. Conclusions

In conclusion, the results of this study demonstrate that postoperative ileus is a common occurrence after spinal fusion for NMS. Abdominal radiographs do not provide useful diagnostic information to change management or assess for a more serious obstructive gastrointestinal pathology. The additional cost and radiation burden of these low-utility exams should be carefully considered. In the presence of postoperative abdominal distension, feeding intolerance, and delayed return of bowel function, we advocate for continuing conservative measures such as bowel rest, maintenance intravenous fluids, nasogastric decompression, and serial abdominal examinations until bowel function returns. Abdominal radiographs and a surgical team consultation should be reserved for patients with a worsening exam despite bowel rest or additional causes for concern, such as guarding or rebound on exam, aberrant laboratory markers or vital signs, or persistent bloating or emesis.

## Figures and Tables

**Table 1:** Characteristics, follow-up, and diagnoses of patients undergoing posterior spinal fusion for neuromuscular scoliosis.

Characteristics	
Age, years (mean ± SD)	13.7 ± 2.6
**Sex**	
Male	76 (57.1%)
Female	57 (42.9%)
BMI, kg/m^2^ (mean ± SD)	18.3 ± 4.4
Preoperative major Cobb, degrees (mean ± SD)	82.8 ± 23.0
Follow-up, months (mean ± SD)	44.7 ± 15.4
**Diagnosis**	
Cerebral palsy	67 (50.4%)
Muscular dystrophy	19 (14.3%)
Myelomeningocele	8 (6.0%)
Spinal muscular atrophy	8 (6.0%)
Other	31 (23.3%)
**Nutrition Status**	
G-tube dependent	62 (46.6%)
No G-tube	71 (53.4%)
**Ambulatory Status**	
Non-ambulatory	97 (72.9%)
Ambulatory	36 (27.1%)

All values are presented as *n* (%) unless otherwise noted. SD = standard deviation.

**Table 2:** Results of abdominal radiographs.

KUB Result		Management
Ileus	15 (50%)	No change in management
Nonspecific bowel dilation	13 (43.3%)	
	11 (36.7%)	No change in management
	2 (6.7%)	Pediatric surgery consultation without change in management
Other	2 (6.7%)	
Fecal impaction	1 (3.3%)	GI consultation and bowel clean out
Pneumatosis of the colon	1 (3.3%)	Pediatric surgery consultation, exploratory laparotomy

All values are presented as *n* (%).

**Table 3:** Comparison of groups with and without KUB performed.

Characteristic	KUB Performed (*N* = 30)	No KUB Performed (*N* = 103)	*p*-Value
Age, years, mean ± SD	13.6 ± 2.7	13.7 ± 2.5	0.783
Sex (male), *n* (%)	15 (50)	61 (59.2)	0.406
Nutrition status (G-tube present), *n* (%)	15 (50)	47 (45.6)	0.684
Ambulatory status (Non-ambulatory), *n* (%)	26 (86.7)	71 (68.9)	0.064
BMI, kg/m^2^, mean ± SD	19.0 ± 4.6	18.1 ± 4.3	0.316
Preop Cobb, degrees, mean ± SD	87.4 ± 23.6	81.5 ± 22.7	0.225
Change in Cobb, degrees, mean ± SD	59.9 ± 18.1	54.6 ± 18.8	0.188
EBL, mL, mean ± SD	907 ± 515	743 ± 440	0.092
OR duration, minutes, mean ± SD	450 ± 117	375 ± 128	* 0.005

All values are presented as *n* (%) unless otherwise noted. SD = standard deviation. * indicates statistical significance (*p* value < 0.05).

## Data Availability

The data that support the findings of this study are available from the corresponding author, TW, upon reasonable request.
